# The Values of Combined and Sub-Stratified Imaging Scores with Ultrasonography and Mammography in Breast Cancer Subtypes

**DOI:** 10.1371/journal.pone.0145390

**Published:** 2015-12-21

**Authors:** Tsun-Hou Chang, Hsian-He Hsu, Yu-Ching Chou, Jyh-Cherng Yu, Giu-Cheng Hsu, Guo-Shu Huang, Guo-Shiou Liao

**Affiliations:** 1 Department of Radiology, Tri-Services General Hospital, Taipei, Taiwan; 2 Department of Radiology, National Defense Medical Center, Taipei, Taiwan; 3 School of Public Health, National Defense Medical Center, Taipei, Taiwan; 4 Division of General Surgery, Department of Surgery, Tri-Services General Hospital, National Defense Medical Center, Taipei, Taiwan; 5 Breast Medical Center, Kang-Ning General Hospital, Taipei, Taiwan; School of Medicine, Fu Jen Catholic University, TAIWAN

## Abstract

**Background and Objectives:**

The Breast Imaging Reporting and Data System (BI-RADS) of Mammography (MG) and Ultrasonography (US) were equivalent to the “5-point score” and applied for combined and sub-stratified imaging assessments. This study evaluated the value of combined and sub-stratified imaging assessments with MG and US over breast cancer subtypes (BCS).

**Materials and Methods:**

Medical records of 5,037 cases having imaging-guided core biopsy, performed from 2009 to 2012, were retrospectively reviewed. This study selected 1,995 cases (1,457 benign and 538 invasive cancer) having both MG and US before biopsy. These cases were categorized with the “5-point score” for their MG and US, and applied for combined and sub-stratified imaging assessments. Invasive cancers were classified on the basis of BCS, and correlated with combined and sub-stratified imaging assessments.

**Results:**

These selected cases were evaluated by the “5-point score.” MG, US, and combined and sub-stratified imaging assessments all revealed statistically significant (P < 0.001) incidence of malignancy. The sensitivity was increased in the combined imaging score (99.8%), and the specificity was increased in the sub-stratified combined score (75.4%). In the sub-stratified combined imaging assessment, all BCS can be classified with higher scores (abnormality hierarchy), and luminal B subtype showed the most salient result (hierarchy: higher, 95%; lower, 5%).

**Conclusions:**

Combined and sub-stratified imaging assessments can increase sensitivity and specificity of breast cancer diagnosis, respectively, and Luminal B subtype shows the best identification by sub-stratified combined imaging scoring.

## Introduction

Mammography (MG) has been shown to reduce mortality from breast cancer, and ultrasonography (US) is a well-known adjunct to screening MG [1–4]. The American College of Radiology (ACR) Breast Imaging Reporting and Data System (ACR BI-RADS^®^) provides standardized descriptors of imaging features of breast lesions, irrespective of the modality—MG, US, or magnetic resonance imaging (MRI); it is also helpful in predicting benign or malignant potential, and can be used globally. The latest edition of ACR BI-RADS^®^ was announced in late 2013 [5]. Recently, many studies have been discussing “the importance of US” on screening or diagnostic scenarios [6–8], but few articles have discussed combined MG and US [9].

The Royal College of Radiologists’ Breast Group, United Kingdom (RCR-UK; now rename as British Society of Breast Radiology) provided a 5-point scoring system, which was first described in 1998 and formalized by Maxwell et al [10, 11]. This scoring system was quantified and mapped to ACR BI-RADS^®^ by Taylor et al [12] in 2011. Wilkinson et al commented that this scoring system was being used for communication across the multidisciplinary team with analogous systems for clinical examination, MRI, cytology, and histopathology reporting [13]. The differences between ACR BI-RADS^®^ and RCR-UK 5-point scoring system are that latter can be applied on histopathology results and combined use with triple assessment over the diagnostic cases [13].

ACR BI-RADS^®^ classification is actually more practical than RCR-UK 5-point scoring system. However, more published literatures [9–13] have discussed RCR-UK 5-point scoring system correlation with histopathology and combined uses with triple assessment (examination, imaging, and biopsy) on the palpable diagnostic cases.

In our hospital, breast radiologists and surgeons conduct a combined assessment using MG and US features to predict the likelihood of cancer for the patients with a palpable breast mass. The assessment system is simply based on the hierarchy of ACR BI-RADS^®^ categories of each MG and US respectively. More diagnostic cases need composite reports with MG and US to make a final diagnosis.

The proposed classification, by the St Gallen International Breast Cancer Conference 2011, into molecular subtypes when routine biomarker analysis by IHC is used as a surrogate for genetic analysis, includes luminal A and B, luminal human epidermal growth factor receptor 2 (HER2), HER2 overexpression, and triple negative (TN) [14]. Distinct molecular subtypes respond differently to therapy [7, 15–18] and have different prognoses [19–22].

Our main aim was to verify the combined and sub-stratified imaging assessments using MG and US over diagnostic cases. We also aimed to investigate the relationships among breast cancer subtypes (BCS) in the combined and sub-stratified imaging assessments.

## Materials and Methods

### Study population

**Fig 1:**
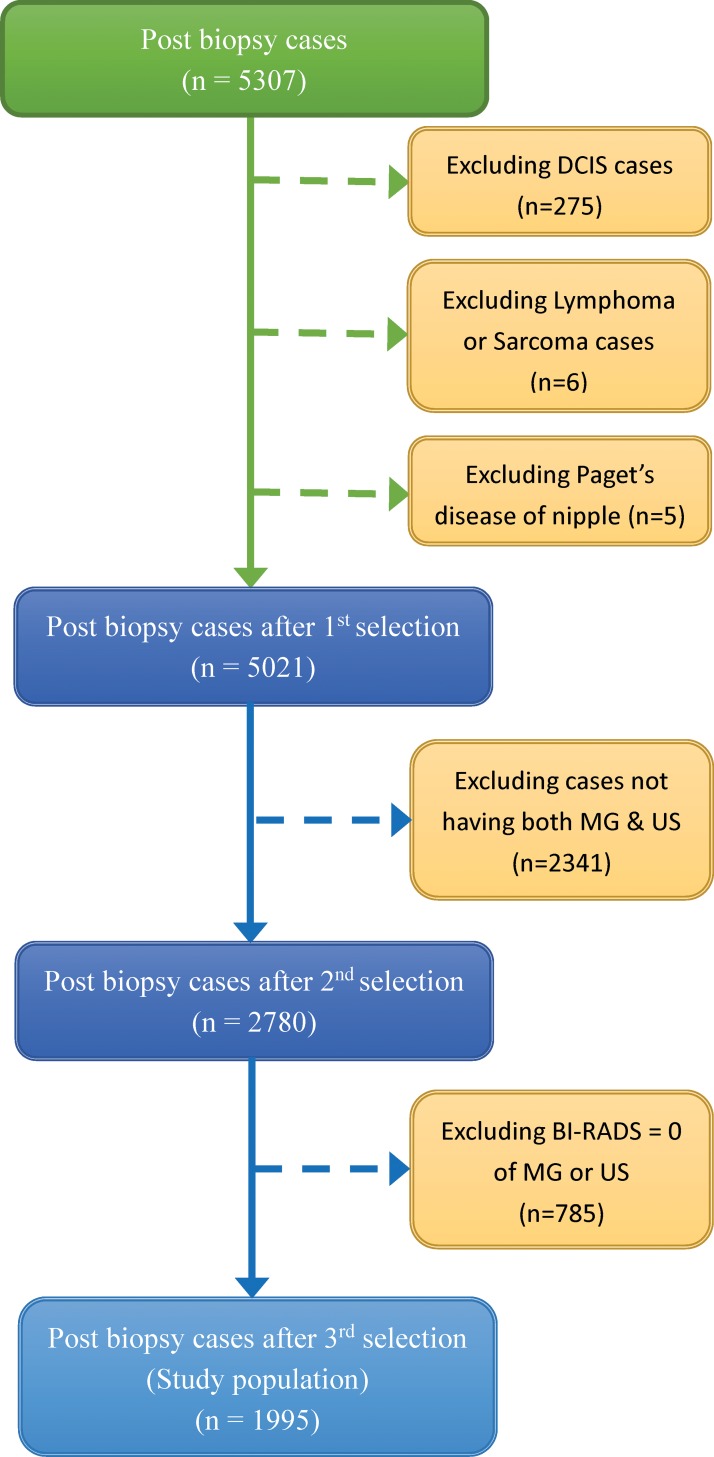
Flow chart of summarizing patient selection.

This study was approved by Institutional Review Board (IRB) of Tri-Service General Hospital (TSGHIRB No: 1-103-05-110); informed consent was waived as the data were analyzed anonymously and retrospectively. We reviewed the medical records of post-core-needle or post-surgical biopsy cases in Tri-Service General Hospital from January 2009 to December 2012, amounting to 5,307 consecutive post-biopsy cases. The inclusion criteria were all biopsy cases before any clinical treatment. We excluded cases with Paget’s disease of the nipple, DCIS, breast lymphoma, or sarcoma, as well as those in which both MG and US were not performed. In addition, we also excluded the cases with ACR BI-RADS category 0 of MG or US. Finally, totally 1,995 cases were finally selected for this study (Fig 1). The subset of patients, comprising 1,457 benign cases and 538 malignant cases, was included for analysis. If the patients underwent more than one imaging examination before tissue biopsy, the latest one was analyzed. In patients with bilateral biopsies or more than one biopsy in one breast, the most serious result was considered.

### Imaging protocols

There are two digital MG machines in our institution, both with full-field digital mammograms (Hologic-Lorad Inc. Bedford, MA, USA). Diagnostic mammograms were obtained using standard craniocaudal (CC) and mediolateral oblique (MLO) views by well-trained technologists, and the findings were reported by four experienced radiologists (5, 8, 15, and 20 years’ experience in breast imaging).

All US examinations included real-time bilateral whole-breast and power Doppler blood flow scans, using three US machines (GE Medical System, Milwaukee, WI, USA). Two are Logiq P6 and one is Logiq L7 with 7–12-MHz probes. US was performed by experienced technologists, and the findings were reported by on-duty radiologists under ACR BI-RADS categories.

MG and US can supplement each other. The physician made an ensemble decision from MG and US reports before deciding on biopsy.

### Data analysis

We modified the RCR-UK 5-point scoring system for all selected cases (Table 1). Scores (categories) 1 and 2 have the same definitions in ACR BI-RADS^®^ and RCR-UK 5-point scoring system, and we did not modify this. The major difference was in the definition of score 3: it is “probably benign” in BI-RADS^®^ and “indeterminate or probably benign” in RCR-UK, and we modified it to “indeterminate, probably benign or low suspicious” in our scoring system. We classified BI-RADS^®^ categories 3 and 4a as “score 3” and BI-RADS^®^ category 4b and 4c as “score 4”; “score 5” was same as BI-RADS^®^ category 5.

**Table 1:** Comparison of Our 5-Point Scoring and RCR-UK* 5-Point Classification Equivalent to BI-RADS**.

Our institute 5-point scoring		RCR 5-point classification	
Score and definition	BI-RADS	Score and definition	BI-RADS
1.Normal without abnormal findings	1	1.Normal	1, 2
2.Benign abnormal findings	2	2.Benign	3
3.Probably benign or low suspicion	3, 4a	3.Indeterminate / probably benign findings	4a, 4b
4.Moderate or high suspicion for malignancy	4b, 4c	4.Finding suspicious of malignancy	4c
5.Highly suspicious of malignancy	5	5.Finding highly suspicious of malignancy	5

* RCR-UK = Royal College of Radiologists Breast Group of United Kingdom

** BI-RADS = Breast Imaging Reporting and Data System

All cases’ malignant pathological results were used to classify the BCS: luminal A (estrogen receptor (ER) + and/or progesterone receptor (PR)+, low or intermediate grade, and HER2–), luminal B (ER+ and/or PR+, high grade, and HER2–), luminal HER2+ (ER+ and/or PR+, any grade, and HER2+), HER2 overexpression (ER–, PR–, any grade, and HER2+), and triple negative (ER–, PR–, any grade, and HER2–)[14, 23]. The definition of ER/PR positivity was determined by IHC of the number of positively stained nuclei (>1%, +) [24]. Tumors were considered as HER2 positive when cells exhibited a strong membrane staining (3+) for HER2 protein overexpression; 0 or 1+ were considered as HER2 negative, and in cases of equivocal membrane staining (DAKO score 2+), fluorescence in situ hybridization (FISH) was used to evaluate gene amplification [25].

All selected cases were re-evaluated by our 5-point score with the following assessments: (1) MG alone, (2) US alone, (3) combined MG and US, and (4) sub-stratified combined score with MG and US. In the combined imaging assessment, the higher BI-RADS category was considered as the score. In the sub-stratified combined imaging assessment, each score 3, 4, and 5 were subdivided into three subgroups of a, b, and c. For the MG alone, US alone, and combined imaging scores, scores 1 and 2 indicated negative for cancer and scores 3–5 indicated positive for cancer. For the sub-stratified combined scores, scores 1–3b were regarded as negative for cancer and 3c–5 as positive for cancer.

### Statistical analysis

All statistical analyses were performed using PASW statistical software (ver. 18.0; SPSS, Inc., Chicago, IL). The chi-square test and Fisher exact test were used to compare the distribution of MG alone, US alone, the combined image score, the sub-stratified combined score. The sensitivity, specificity, positive predictive value, and negative predictive value for each assessment was calculated. The relationships between BCS and combined imaging and sub-stratified imaging assessments were also done. The P values were two-sided and were considered statistically significant when less than 0.05.

## Results

Of 5,307 consecutive post-biopsy cases, the histopathology of Paget’s disease of the nipple (n = 5), DCIS (n = 275), breast lymphoma (n = 4), or sarcoma (n = 2) were excluded in the first step. Then there were 2,341 cases without both having MG and US, and 785 cases with ACR BI-RADS category 0 of MG or US. They were excluded in the patient selection (Fig 1). A total 1,995 cases were selected in the study population (age range, 25–95 years; mean age, 48 ± 12 years). In Table 2, malignancy incidence for each group of imaging assessment revealed as follows: MG alone score; US alone score; combined imaging score; and sub-stratified combined score. Eleven cases with combined imaging score of 2 underwent biopsy because of unknown nipple discharge, prophylactic excision due to breast cancer family history, and removal of palpable “mass” according to the patient’s request. Among them, 1 (9.1%) had a cancer diagnosis. For cases with sub-stratified combined imaging scores of 3a, 3b, and 3c, the malignancy incidence was 2.5%, 6.7%, and 12.3%, respectively. Malignant incidence of sub-stratified combined assessment also increased with higher scores, which are similar to Li’s results [9].

**Table 2:** Case of Malignancy Incidence for Each Group of Categories.

Variable assessment group	Benign (%)	Malignancy (%)	No of patient	P value
**Mammography (MG) alone**				<0.001*
1.Normal without abnormal findings	514(88.8)	65(11.2)	579	
2.Benign abnormal findings	570(88.9	71(11.1)	641	
3.Indeterminate or uncertain	306(85.2)	53(14.8)	359	
4.Suspicious of malignancy	62(21.8)	223(78.2)	285	
5.Highly suspicious of malignancy	5(3.8)	126(96.2)	131	
Total	1,457(73.0)	538(27.0)	1,995	
**Ultrasonography (US) alone**				<0.001*
1.Normal without abnormal findings	7(77.8)	2(22.2)	9	
2.Benign abnormal findings	84(95.5)	4(4.5)	88	
3.Indeterminate or uncertain	1,240(91.0)	123(9.0)	1,363	
4.Suspicious of malignancy	121(33.1)	245(66.9)	366	
5.Highly suspicious of malignancy	5(3.0)	164(97.0)	169	
Total	1,457(73.0)	538(27.0)	1,995	
**Combined Scoring with MG & US**				<0.001*
1.Normal without abnormal findings	0(0.0)	0(0.0)	0	
2.Benign abnormal findings	10(90.9)	1(9.1)	11	
3.Indeterminate or uncertain	1,295(92.6)	103(7.4)	1,398	
4.Suspicious of malignancy	144(37.4)	241(62.6)	385	
5.Highly suspicious of malignancy	8(4.0)	193(96.0)	201	
Total	1,457(73.0)	538(27.0)	1,995	
**Sub-Stratified Combined Scoring with MG & US**				
				<0.001*
1. Score 1	0(0.0)	0(0.0)	0	
2. Score 2	10(90.9)	1(0.0)	11	
3. Sore 3				
3a. MG 3 + US 1 or 2	77(97.5)	2(2.5)	79	
3b. US 3 + MG 1 or 2	1011(93.4)	72(6.6)	1083	
3c. MG 3 + US 3	207(87.7)	29(12.3)	236	
4. Score 4				
4a. MG 4 + US 1, 2 or 3	24(54.5)	20(45.5)	44	
4b. US 4 + MG 1, 2 or 3	84(45.6)	67(44.4)	151	
4c. MG 4 + US 4	36(18.9)	154(81.1)	190	
5. Score 5				
5a. MG 5 + US 1 to 4	3(9.4)	29(90.6)	32	
5b. US 5 + MG 1 to 4	3(4.3)	67(95.7)	70	
5c. MG 5 + US 5	2(2.0)	97(98.0)	99	
Total	1,457(73.0)	538(27.0)	1,995	

1. Original BI-RADS categories have been modified to the RCR 5-point scoring.

2. All assessments were made with a combination of MG and US. The category was determined by the higher score from MG and US.

3. P value from two-sided Chi Square test.

*Statistically significant.

The sensitivity of combined imaging assessment was the highest (99.8%), and the specificity was the highest in the sub-stratified combined imaging assessment (75.4%) (Table 3). The positive predictive value was the highest with the sub-stratified combined score, and negative predictive value was the highest with the US alone score.

**Table 3:** Comparison of Results by Different Assessment Methods.

Variable	Sensitivity (%)	Specificity (%)	Positive Predictive Value (%)	Negative Predictive Value (%)
**Mammography (MG) alone**	74.7	74.4	51.9	88.9
**Ultrasonography (US) alone**	98.9	6.2	28.0	93.8
**Combined Scoring with MG and US**	99.8	0.7	27.1	90.9
**Sub-Stratified Combined Scoring with MG and US**	86.1	75.4	56.3	93.6

The sub-stratified combined score can be divided into two groups (lower hierarchy: score 1–3b; higher hierarchy: score 3c–5), and to correlate with BCS (Table 4). The results revealed all BCS can be classified with higher hierarchy of each imaging assessment. Luminal B subtype showed the most salient result (hierarchy: higher, 95%; lower, 5%), but luminal A subtype revealed less difference in the sub-stratified combined imaging assessment.

**Table 4:** Comparison of Sub-Stratified and Combined Scoring in the Breast Cancer Subtypes.

Variable	Luminal A (%) (n = 220)	Luminal B (%) (n = 139)	Luminal HER2 (%) (n = 90)	HER2 (%) (n = 54)	TN (%) (n = 19)	P value
**Combined Scoring with MG and US**						1.00
Score 1–2	1(0.5)	0(0)	0(0)	0(0)	0(0)	
Score 3–5	219(99.5)	139(100.0)	90(100.0)	54(100.0)	19(100.0)	
**Sub-Stratified Combined Scoring with MG and US**						0.002*
Score 1–3b	43(19.5)	7(5.0)	12(13.3)	8(14.8)	2(10.5)	
Score 3c–5	177(80.5)	132(95.0)	78(86.7)	46(85.2)	17(89.5)	

HER2 = Human Epidermal growth factor Receptor 2 overexpression subtype; TN = Triple Negative subtype; P value from two-sided Chi Square test.

*Statistically significant.

## Discussion

In clinical practice, only negative MG or US cannot totally exclude the possibility of malignancy; this is so-called “false negative” on an imaging test [1, 26]. Our 5-point scoring system applying on the four different assessments (MG alone, US alone, combined score, sub-stratified combined score) indicate different malignancy incidence rates (Table 2), with scores over 3 suggesting the necessity of biopsy. Combined imaging scores show fewer cases of false negative, especially the sub-stratified combined score 3a reveals the least malignant rate (2.5%), which is similar to the previous study by Li et al. [9], the sub-stratified combined assessment revealed the highest specificity of malignant detection, but lower sensitivity than combined imaging assessment (Table 3). Combined imaging modalities are better use than single imaging modality.

Chan et al. [1] indicated that the sensitivity of US alone score was 91%, which was higher than that of MG alone score was 78% [1]. These results are similar to ours (Table 3), with the respective values being 98.9% and 74.7%. In the sub-stratified combined assessment, scores 3c–5 were considered to be positive for cancer, the specificity significantly increased from 0.7% to 75.4%, while the sensitivity changed from 98.9% to 86.1%, indicating very high sensitivity and low specificity. This might be because our institute is a tertiary medical center, where many patients were referred to for further management, and many women with anxiety requested preventive biopsy.

Combined imaging score presented more salient results than US alone. There is a higher percentage of dense breasts in Asian women, and US has been a routine, supplemental modality to examine a “palpable mass.” Therefore, many cases were false positive [6, 27].

Of the two combined imaging assessments, the sub-stratified combined score has the higher positive and negative predictive values and the combined imaging score showed the best sensitivity (Table 3).

Breast cancer is a heterogeneous group of neoplasms with multivariate morphology, growth pattern, molecular profiles, and response to treatment [28, 29]. BCS are important and defined according to some specific IHC markers. Based on these qualities of the specificity and sensitivity of the sub-stratified combined and combined imaging scores, we observed that sub-stratified combined assessment showed more significant and correlation with BCS. In the sub-stratified combined assessment, more suspicious malignant assessments (higher score) had higher percentage of luminal B, TN, luminal HER2, or HER2 overexpression subtypes, except for luminal A subtype (Table 4). Luminal B subtype exhibits more high-grade cancer cells than any other BCS, luminal A subtype exhibits more low- to intermediate-grade cancer cells, and the other subtypes do not have limits on cell grades (any grade) [14, 23]. This can explain why more cancers of luminal A subtype were still identified with lower hierarchy of scoring (benign assessments), but luminal B subtype revealed the most cases with higher hierarchy of scoring (Table 4). Studies discussing correlation of imaging findings (e.g., multifocal lesions, shape, lymph node involvement) with BCS [30–33] reported that high-grade cancers or cancers with poorly prognosis present with more additional suspicious findings on breast imaging [30, 31, 33, 34].

To our knowledge, ours is the first report to correlate BCS with combined MG and US assessments. We know that many suspicious features may not certainly positively correlation with assessing scoring. This is why we cannot tell imaging scoring from these BCS clearly, but a trend of high-grade cancer cells may be related to higher hierarchy of imaging scoring (such as luminal A and B subtypes in our data).

The main limitation of our study was patient selection. We retrospective selected only post-biopsy cases, which may have a higher risk of malignancy than the general population. Further, the cases with scores of 1–3b in the sub-stratified combined method were neither followed up nor subjected to biopsy. In addition, our sample size was relatively small, and larger studies are needed to corroborate our findings with statistical analysis.

## Conclusions

Combined and sub-stratified imaging assessments can increase sensitivity and specificity respectively. There were significant differences between Luminal A and B subtypes in the sub-stratified combined imaging scoring. Luminal B subtype show the best identification by sub-stratified combined imaging scoring.
