# Correction: The Values of Combined and Sub-Stratified Imaging Scores with Ultrasonography and Mammography in Breast Cancer Subtypes

**DOI:** 10.1371/journal.pone.0148005

**Published:** 2016-01-25

**Authors:** Tsun-Hou Chang, Hsian-He Hsu, Yu-Ching Chou, Jyh-Cherng Yu, Giu-Cheng Hsu, Guo-Shu Huang, Guo-Shiou Liao

There is an error in affiliation 1 for author Tsun-Hou Chang. Affiliation 1 should be: Department of Radiology, Tri-Services General Hospital, National Defense Medical Center, Taipei, Taiwan.
